# Sensitivity to Change and Patient‐Centricity of the Unified Multiple System Atrophy Rating Scale Items: A Data‐Driven Analysis

**DOI:** 10.1002/mds.28993

**Published:** 2022-03-25

**Authors:** Florian Krismer, Klaus Seppi, Linus Jönsson, Daniel Oudin Åström, Anna‐Karin Berger, Jacob Simonsen, Mark Forrest Gordon, Gregor K. Wenning, Werner Poewe, European Multiple System Atrophy Study Group Natural History Study Investigators, the Rasagiline‐for‐Multiple System Atrophy Investigators

**Affiliations:** ^1^ Department of Neurology Medical University Innsbruck Innsbruck Austria; ^2^ Section for Neurogeriatrics, Department for Neurobiology, Care and Society Karolinska Institutet Stockholm Sweden; ^3^ H. Lundbeck A/S Valby Denmark; ^4^ Teva Pharmaceuticals West Chester Pennsylvania USA

**Keywords:** multiple system atrophy, clinical outcome assessment, Unified Multiple System Atrophy Rating Scale, patient‐centricity

## Abstract

**Background:**

The Unified Multiple System Atrophy Rating Scale (UMSARS) is a commonly used semiquantitative rating scale to assess symptoms and measure disease progression in multiple system atrophy (MSA). However, it is currently incompletely understood which UMSARS items are the most sensitive to change and most relevant to the patient.

**Objective:**

The objective of this study was to assess sensitivity to change and patient‐centricity of single UMSARS items.

**Methods:**

Data were taken from the European Multiple System Atrophy Study Group Natural History Study and the Rasagiline for Multiple System Atrophy trial. Sensitivity of change of an item of the UMSARS was assessed by calculation of a sensitivity‐to‐change ratio using its mean slope of progression divided by the standard deviation of the slope when modeling its progression over time. Patient‐centricity was assessed through correlation of UMSARS items with quality‐of‐life measures.

**Results:**

Progression rates above the mean in at least one of the two studies examined here were seen for seven items of UMSARS I and 11 items of UMSARS II. These items related to key motor functions such as swallowing, speech, handwriting, cutting food, hygiene, and dressing or walking, whereas items related to autonomic dysfunction were generally less sensitive to change in either data set. More UMSARS I items were identified as patient‐centric compared with UMSARS II items, and items most strongly impacting patients' quality of life were those affecting verbal communication skills, personal hygiene, and walking.

**Conclusion:**

The present results illustrate the potential to optimize the UMSARS to enhance sensitivity to change and patient centricity. © 2022 The Authors. *Movement Disorders* published by Wiley Periodicals LLC on behalf of International Parkinson and Movement Disorder Society

Multiple system atrophy (MSA) is an orphan neurodegenerative disorder characterized clinically by a combination of parkinsonism, cerebellar ataxia, and autonomic failure.^1^ The α‐synuclein–positive glial cytoplasmic inclusions in multiple brain areas are the pathological hallmarks of the disease.^2^ Depending on the predominant clinical presentation, MSA is subdivided into two main motor phenotypes: a parkinsonian variant characterized by an akinetic‐rigid syndrome with poor or transient response to levodopa treatment (MSA‐P) and a cerebellar variant with progressive ataxia (MSA‐C), each of which is, in addition, associated with varying degrees of autonomic failure.^3^ At present, symptomatic treatment options are limited: parkinsonism is usually poorly responsive to dopaminergic treatment, autonomic failure is difficult to control in the longer term, and there are no effective therapies for cerebellar ataxia.^1^ At the same time, MSA is rapidly progressive leading to severe disability within 4 to 7 years of disease onset in most patients, and median survival averages only approximately 9 years.^4^ Therapies that would slow or prevent progression and therefore are a critical unmet need in MSA, but so far none of the putative disease‐modifying therapies tested in controlled clinical trials have proved successful.

Sensitive and clinically relevant outcome measures are a critical design element for disease‐modification trials in neurodegenerative diseases such as MSA. The most commonly used semiquantitative rating scale to assess symptoms and measure disease progression in MSA is the Unified Multiple System Atrophy Rating Scale (UMSARS).^5^ The scale can be administered in reasonably short time and has been shown to be multidimensional and reliable. Cutoffs for minimal clinically meaningful worsening on the UMSARS scale have been recently established.^6^

Although the UMSARS has shown overall sensitivity to change both in natural history studies and clinical trials, different UMSARS items might behave differently, and it is currently unknown which are the most sensitive to change.^7^, ^8^ In this article, we applied a data‐driven approach using the European Multiple System Atrophy Study Group Natural History Study (EMSA NHS)^7^ as well as data from a randomized controlled clinical trial (Rasagiline for Multiple System Atrophy [MSA‐Ras])^9^ to assess the sensitivity to change and the degree of patient centricity of single UMSARS items.

## 1 Methods

### 1.1 Sources of UMSARS Data

The EMSA NHS was a prospective natural history study that has been described in detail elsewhere.^7^ Briefly, 15 European movement disorders centers participated in this study and enrolled patients with a clinical diagnosis of MSA. All patients were interviewed and examined by board‐certified neurologists with specialized experience in movement disorders. Study duration was 2 years with a structured follow‐up visit every 6 months. Validated rating instruments, including the UMSARS, were applied to investigators, patients, or caregivers as appropriate. Disease‐specific symptoms were assessed by the UMSARS and quality of life (QoL) by the Medical Study Short Form survey (SF‐36)^10^ and the five‐dimensional EuroQoL (EQ‐5D).^11^

The MSA‐Ras study (NCT00977665) was a multicenter, randomized, double‐blind, placebo‐controlled study sponsored by Teva Pharmaceutical Industries, Ltd (Netanya, Israel) and H. Lundbeck (Valby, Denmark) that investigated the effects of rasagiline on symptom progression in patients with a diagnosis of possible or probable MSA‐P according to consensus criteria.^9^ The study period was 48 weeks, and inclusion criteria were defined to capture early disease stages (<3 years from the time of documented MSA diagnosis; anticipated survival of at least 3 years; and exclusion of patients with severe orthostatic symptoms, severe impairment of speech, swallowing, and ambulation, and/or more than one fall per week). Clinical progression was assessed at baseline and study weeks 12, 24, 36, and 48 using the UMSARS. The patients' health‐related QoL was measured using the Multiple System Atrophy Quality of Life Questionnaire (MSA‐QoL).^12^ Although treatment with rasagiline 1 mg/day did not show a significant effect on disease progression compared with placebo as assessed by the UMSARS, the study provides detailed longitudinal information on patients with MSA.

### 1.2 Item Structure of the UMSARS

The UMSARS^5^ is divided into four parts comprising a historical, patient interview–based assessment of impairments affecting activities of daily living (UMSARS part I) and physician‐based ratings including motor (UMSARS part II) and autonomic assessments (UMSARS part III) as well as a single item on global disability (UMSARS part IV).^5^ UMSARS I consists of 12 items, and UMSARS II consists of 14 items. Each item is rated from zero to four, with zero corresponding to an unaffected/normal state and four to very severe disability in the patient. Higher scores on the UMSARS reflect greater symptom burden. For the present analysis, only parts I and II were considered.

### 1.3 Statistical Analysis

#### 1.3.1 Sensitivity to Change

The “sensitivity to change” of an item of the UMSARS I or UMSARS II was assessed by calculation of a sensitivity to change ratio (SCS) using its mean slope of progression divided by the standard deviation of the slope when modeling its progression over time with a linear mixed model with random slope and intercept. In the EMSA NHS data set, the sensitivity to change was calculated using 24 months of follow‐up, whereas in the MSA‐RAS it was calculated using 48 weeks of follow‐up. The more “sensitive to change” an item of the UMSARS is (compared with other items of the UMSARS), the lower its rank will be. For illustration purposes, a standard score (*z* score) was calculated.

#### 1.3.2 Patient Centricity

Patient centricity was assessed on the basis of correlation of UMSARS items with QoL measures (either generic QoL scales [EMSA NHS] or a disease‐specific scale [MSA‐Ras]). In the EMSA NHS study, an item of the UMSARS I or II was classified as “patient centric” if it showed a correlation coefficient above 0.5 with at least one of the QoL measurements (EQ‐5D or SF‐36) at three or more time points, including a time point within 12 months of baseline examination. Similarly, in the MSA‐Ras study, an item of the UMSARS I or II was considered “patient‐centric” if it showed a correlation coefficient above 0.5 with one of MSA‐QoL subscales at the baseline or final follow‐up visit.

#### 1.3.3 Optimization

Discrete optimization with binary weights for each item (ie, inclusion weights of zero or one) was performed to find an optimal combination of UMSARS items (ie, an abbreviated UMSARS scale that is patient centric and sensitive to change).

Our measure of sensitivity to change of a subset of items is the standardized sum of changes of single‐item scores restricted to items within the subset. The quantitative measure of sensitivity to change can therefore be expressed as the value of the function *f*, which is defined by: $f\left(w\right)=-\text{mean}\left(\sum_{j=1}^{26}w_{j}\alpha_{\mathrm{ij}}\right)/\mathrm{sd}\left(\sum_{j=1}^{26}w_{j}\alpha_{\mathrm{ij}}\right)$, where *α*_*ij*_ corresponds to the fitted slope of the UMSARS item *j* of patient *i* and the mean and standard deviation run over the patient index *I* and *w*_*j*_ is the item weight for each item, either zero or one. The most “sensitive to change” among all possible combinations of binary weights for all UMSARS item slopes was the combination for which the function *f* is at its maximum. Thus, the subset that had the highest value of this measure is defined as the “most sensitive to change” combination of items of all possible 2^26^ subsets.”

To ensure patient centricity in the abbreviated version, we restricted the second optimization to have binary weights of one for the patient‐centric items and tested all other possible combinations that again minimized the aforementioned function. In addition, we ran an optimization where the UMSARS item weights were allowed to be continuous.

## 2 Results

### 2.1 Demographics

The two study populations were similar regarding sex distribution and class of diagnostic certainty (possible vs. probable). Symptom duration at baseline was significantly longer and age at baseline was significantly lower in the EMSA NHS compared with the MSA‐Ras trial. Also, the MSA‐Ras trial only included patients with MSA‐P, whereas the EMSA NHS recruited patients with both MSA‐P and MSA‐C. Further baseline characteristics of the study population are shown in Table 1.

**TABLE 1:** Demographics

Variables	EMSA NHS, N = 150	MSA‐Ras, N = 174	*P* value
Sex, n (%)			0.884^a^
Male	85 (56.7)	100 (57.5)	
Female	65 (43.3)	74 (42.5)	
Symptom duration, baseline, mean (SD)	5.49 (3.95)	3.93 (2.37)	<0.001^b^
Age at baseline, mean (SD)	63.05 (8.24)	65.02 (8.49)	0.036^b^
Predominant motor presentation, n (%)			<0.001^a^
MSA‐C	55 (36.7)	0 (0.0)	
MSA‐P	95 (63.3)	174 (100.0)	
Diagnostic certainty, n (%)			0.921^a^
Possible	81 (54.0)	93 (53.4)	
Probable	69 (46.0)	81 (46.6)	

Abbreviations: EMSA NHS, European Multiple System Atrophy Natural History Study; MSA‐Ras, Rasagiline for Multiple System Atrophy; SD, standard deviation; MSA‐C, multiple system atrophy cerebellar variant with progressive ataxia; MSA‐P, multiple system atrophy akinetic‐rigid syndrome with poor or transient response to levodopa treatment.

^a^ Pearson's chi‐squared test.

^b^ Linear model analysis of variance.

### 2.2 UMSARS Item Sensitivity to Change and Patient Centricity

**FIG 1:**
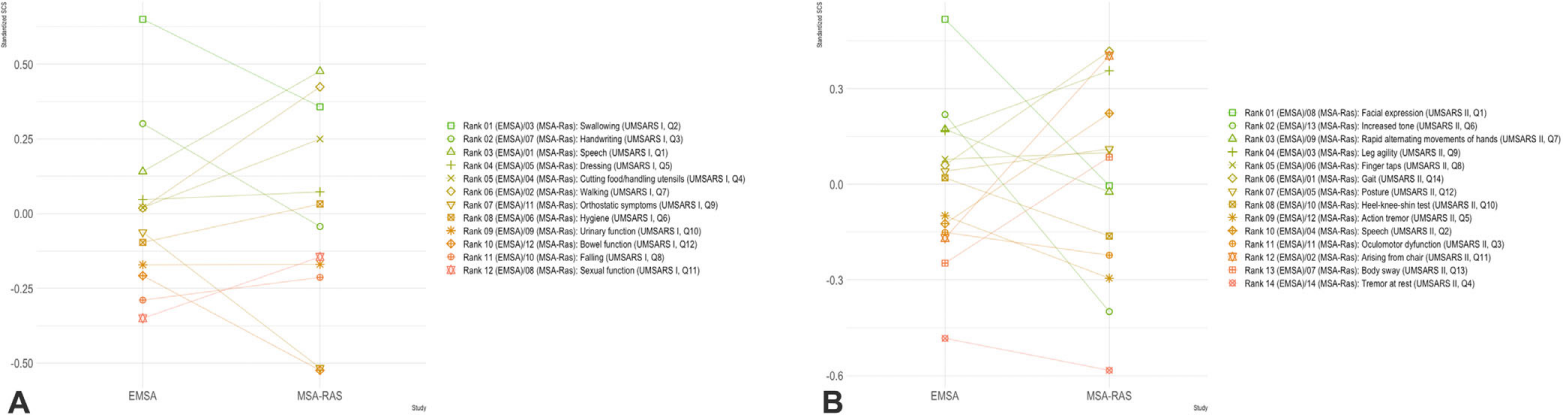
Comparison of standardized change scores between different studies. EMSA, European Multiple System Atrophy Study Group; MSA‐Ras, Rasagiline for Multiple System Atrophy Trial; SCS, standardized change score expressed as standard score; UMSARS, Unified Multiple System Atrophy Rating Scale. [Color figure can be viewed at wileyonlinelibrary.com]

Progression rates above the mean (ie, a *z* score above 0) in at least one of the two studies were seen for seven items of UMSARS I and 11 items of UMSARS II (see Fig. 1). For UMSARS I, these mainly included items related to key motor functions such as swallowing, speech, handwriting, cutting food, hygiene, dressing, or walking, whereas items related to autonomic dysfunction were generally less sensitive to change in either data set. The latter was also true for the respective items in UMSARS II, and the most sensitive items were those related to parkinsonian motor symptoms. Although five of the seven UMSARS I items with progression rates above the mean in either study were consistent between both data sets, only four of the most sensitive items of UMSARS II had progression rates above the mean in both studies. Importantly, the ranking of the sensitivity to change for 1 and 2 years of follow‐up in the EMSA‐NHS were similar.

From the correlation analyses between items on the UMSARS scale and the QoL scales in the EMSA NHS, more UMSARS I items were identified as patient centric compared with UMSARS II items: these included item 2 (“speech”), item 5 (“dressing”), item 6 (“hygiene”), and item 7 (“walking”) from UMSARS I, whereas for UMSARS II only item 14 (“gait”) met the patient‐centricity criterion. At the baseline visit of the MSA‐Ras study, UMSARS I item 4 (“cutting food and handling utensils”), item 5 (“dressing”), and item 6 (“hygiene”) fulfilled the criteria for patient centricity, whereas item 2 (“speech”) and item 7 (“walking”) were not highly correlated with the MSA‐QoL score. At the end of the study, a total of six UMSARS I items (item 2 [“speech”], item 3 [“handwriting”], item 4 [“cutting food and handling utensils”], item 5 [“dressing”], item 6 [“hygiene”], and item 7 [“walking”]) showed a correlation coefficient of above 0.5. Notably, these items included all four UMSARS I items that correlated with QoL measures in the EMSA NHS as well as lending further support to their validity regarding patient centricity. None of the motor examination (UMSARS II) items met criteria for patient centricity at the baseline visit of the MSA‐RAS study, but at week 48, item 2 (“speech”), item 9 (“leg agility”), item 11 (“arising from chair”), item 13 (“body sway”), and item 14 (“gait”) were associated with health‐related QoL.

### 2.3 UMSARS Scale Optimization Potential

Based on the binary optimization derived from the analysis of EMSA NHS data, eight items were included in the most sensitive‐to‐change version of an abbreviated UMSARS: UMSARS I items 2 (“swallowing”) and 9 (“orthostatic symptoms”) and UMSARS II item 1 (“facial expression”), item 5 (“action tremor”), item 6 (“increased tone”), item 11 (“arising from chair”), item 12 (“posture”), and item 14 (“gait”). Allowing the weights to be continuous, the same items as in the binary approach were identified as having the highest weights. When adding patient centricity as an additional constraint to the abbreviated version, that is, forcing a patient‐centric item to be included, 19 of the original 26 items were included. Details of the abbreviated UMSARS derivates are presented in Table 2 (optimization performed without constraints) and Table 3 (constrained optimization that requires inclusion of patient‐centric items).

**TABLE 2:** Results of the unconstrained optimization used to derive an abbreviated UMSARS

UMSARS item		Slope (STD)	SCS	Rank
Part I				
Swallowing^a^	UMSARS I, item 2	0.55 (0.17)	3.30	3
Orthostatic symptoms	UMSARS I, item 9	0.18 (0.12)	1.50	20
Part II				
Facial expression	UMSARS II, item 1	0.55 (0.13)	4.10	1
Action tremor	UMSARS II, item 5	0.24 (0.10)	2.32	11
Increased tone	UMSARS II, item 6	0.50 (0.15)	3.31	2
Arising from chair	UMSARS II, item 11	0.65 (0.32)	2.01	15
Posture	UMSARS II, item 12	0.48 (0.17)	2.78	7
Gait^a^	UMSARS II, item 14	0.36 (0.13)	2.77	8

Optimization was performed without constraints on abbreviated scale having to contain certain items. Slope indicates average estimate of the slope; rank indicates rank of item sensitivity to change, that is, highest ratio equals highest rank.

Abbreviations: UMSARS, Unified Multiple System Atrophy Rating Scale; STD, standard deviation of slope; SCS, sensitivity to change ratio (slope divided by standard deviation).

^a^ Patient‐centric item as identified by correlation analyses between single items of the UMSARS and quality‐of‐life scales.

**TABLE 3:** Results of the constrained (patient‐centric) optimization used to derive an abbreviated UMSARS

UMSARS item		Slope (STD)	SCS	Rank
Part I				
Swallowing^a^	UMSASR I, item 2	0.55 (0.17)	3.30	3
Handwriting	UMSARS I, item 3	0.36 (0.14)	2.57	9
Dressing^a^	UMSARS I, item 5	0.54 (0.30)	1.78	16
Hygiene^a^	UMSARS I, item 6	0.54 (0.38)	1.42	21
Walking^a^	UMSARS I, item 7	0.37 (0.21)	1.74	17
Orthostatic symptoms	UMSARS I, item 9	0.18 (0.12)	1.50	20
Urinary symptoms	UMSARS I, item 10	0.36 (0.30)	1.21	22
Bowel function	UMSARS I, item 12	0.26 (0.24)	1.10	23
Part II				
Facial expression	UMSARS II, item 1	0.55 (0.13)	4.10	1
Ocular motor dysfunction	UMSARS II, item 3	0.44 (0.21)	2.04	14
Action tremor	UMSARS II, item 5	0.24 (0.10)	2.32	11
Increased tone	UMSARS II, item 6	0.50 (0.15)	3.31	2
Rapid alternating movement hands	UMSARS II, item 7	0.44 (0.14)	3.09	4
Finger taps	UMSARS II, item 8	0.47 (0.17)	2.79	6
Leg agility	UMSARS II, item 9	0.51 (0.17)	2.97	5
Heel–knee–shin test	UMSARS II, item 10	0.63 (0.25)	2.57	10
Arising from chair	UMSARS II, item 11	0.65 (0.32)	2.01	15
Posture	UMSARS II, item 12	0.48 (0.17)	2.78	7
Gait^a^	UMSARS II, item 14	0.36 (0.13)	2.77	8

Optimization procedure forced to include the patient‐centric items in the abbreviated scale. Slope indicates average estimate of the slope; rank indicates rank of item sensitivity to change, that is, highest ratio equals highest rank.

Abbreviations: UMSARS, Unified Multiple System Atrophy Rating Scale; STD, standard deviation of slope; SCS, sensitivity to change ratio (slope divided by standard deviation).

^a^ Patient‐centric item as identified by correlation analyses between single items of the UMSARS and quality‐of‐life scales.

## 3 Discussion

We were able to demonstrate that individual UMSARS items behave differently regarding their sensitivity to change and patient centricity. Although direct comparison of independent data sets is hampered by differences in overall progression rates, we were also able to evaluate between‐study heterogeneity by calculating an SCS ratio (ie, dividing the slope of change by the standard deviation). The mean change of an outcome measure over time divided by the standard deviation of this change is also referred to as the standardized response mean.^13^ This is an important and useful measure of the ability of the outcome measure to detect differences in disease progression between patient subgroups or to detect a treatment effect in the context of a randomized clinical trial. It does not, however, convey any information about the clinical importance or relevance to patients of the measured effect. Following normalization, the relative ranks can be exploited to compare the magnitude of the sensitivity to change in different data sets. Nine of the 26 items in UMSARS I (n = 5) and II (n = 4) consistently ranked among the top items with regard to their SCS score in both data sets examined here. An additional nine items ranked in the top half of the most sensitive‐to‐change items in one of the two data sets. Although many of the items assessing motor and bulbar symptoms were sensitive to change, autonomic symptoms (including orthostatic as well as urinary and bowel symptoms) did not change substantially over time. This is not surprising because one of the key diagnostic requirements for MSA is the presence of severe autonomic failure, and a ceiling effect is to be expected. In addition, autonomic symptoms are amenable to symptomatic treatments that may substantially influence symptom progression over time. Tremor items and oculomotor dysfunction were also among those items that were insensitive to change in the present data sets. The former can be explained by the fact that rest tremor is uncommon in patients with MSA (UMSARS II, item 4), and the latter might be partially explained by an inaccurate description of anchors of the response options.

Intriguingly, the majority of items that were shown to be sensitive to change in at least one of the present data sets were also reported to be sensitive to change in two other independent cohorts.^14^, ^15^ In detail, items 1 to 7 of the UMSARS I and items 2, 9, 11, 12, and 14 of UMSARS II showed overlapping sensitivity to change in more than two independent data sets.

Despite the consistency between our and other studies regarding items found to be the most sensitive to change, there was also considerable heterogeneity between the two data sets examined here with only few items behaving uniformly in our analysis (Fig. 1). The reasons for this variance likely include differences in baseline characteristics such as age, disease duration, or MSA subtype, and it is conceivable that even within the two main motor phenotypes there may be MSA endophenotypes with different rates of progression. Furthermore, symptom duration at baseline visit may be particularly relevant because UMSARS progression may be nonlinear with faster decline in early versus later disease stages as observed in the EMSA NHS.^7^ In addition, patients with MSA presenting with cerebellar signs or autonomic failure have shown a more rapid decline compared with those presenting with parkinsonism in previous cohort studies^4^, ^7^, ^8^, ^16^, ^17^, ^18^ and, hence, differences in the motor and nonmotor symptoms could also partially explain the present observations. Unfortunately, our analysis cannot answer the question if item sensitivities differ between MSA subtypes or different disease stages, and further research is needed to investigate these important issues.

Ceiling effects of the scale itself could be another explanation for the observed differences with EMSA NHS patients being more severely affected at baseline compared with MSA‐Ras patients. Problems with the scale itself relating to psychometric properties with improvable item constructions, including inaccurate descriptions, could be another cause for the observed inconsistencies.

Beyond the sensitivity to change, the utility of a clinical outcome measure in clinical trials is also determined by its relevance to patient function. The US Food and Drug Administration has provided methodological guidance on the principles of patient‐focused drug development to ensure that patients' experiences and perspectives are captured and meaningfully incorporated into drug development processes.^19^ There are concerns that clinical rating instruments of motor performance such as the Unified Parkinson's Disease Rating Scale in Parkinson's disease^20^ or the UMSARS in MSA^5^ may not provide sufficient information on changes in function that are truly relevant to performance and disabilities of patients' activities of daily living. While acknowledging the lack of a consistent formal definition, we have chosen a concept of patient centricity of UMSARS items based on correlation analyses with patient‐reported QoL measurements. Our results suggest that items most strongly correlated with patients' QoL were those affecting verbal communication skills, personal hygiene, and walking. The two data sets examined here provided consistent data for patient centricity of different UMSARS items. All of the items meeting patient‐centricity criteria in the EMSA NHS were also strongly correlated with the QoL at the follow‐up visit of the MSA‐Ras study. Notably, in the MSA‐Ras study, the MSA‐QoL was used to evaluate QoL. The fact that the MSA‐specific QoL scale applied in the MSA‐Ras study was developed based on comprehensive patient scoping interviews and patient feedback rounds supports the relevance of correlating item behavior with QoL measurements as a reasonable approach to capture patient centricity. We acknowledge, however, that within the EMSA study we might have missed relevant patient‐centricity information through the application of generic QoL scales and that the approach used for the present analysis is a surrogate for a direct assessment of patient perceptions. Formal evaluation of patient centricity through direct involvement of patients and their caregivers remains a research priority to ensure that patients' experiences, perspectives, and needs are adequately and comprehensively captured in future versions of MSA rating scales.

The present results illustrate the potential to optimize the UMSARS scale to enhance sensitivity to change and patient centricity. Using a data‐driven approach, our analyses lead to a proposal for an abbreviated UMSARS scale that includes 19 (of the original 26) items that impact patient QoL and are sensitive to detect change over time (Table 3).

Although the clinimetric properties and the utility of this UMSARS version will still have to be tested, the present results can be a helpful source of information for ongoing UMSARS revision efforts. Future collaborations sharing data of different clinical trials and other longitudinal academic studies are warranted to further characterize predictors of differing rates of item progression and correlations with patient‐reported outcomes.

## Financial Disclosures

F.K. reports receiving personal fees from Institut de Recherches Internationales Servier, Clarion Healthcare, and the Austrian Society of Neurology and grant support from the MSA Coalition outside of the submitted work. K.S. reports personal fees from Teva, UCB S.A., Lundbeck , AOP Orphan Pharmaceuticals AG, Roche, Grünenthal, and AbbVie; honoraria from the International Parkinson and Movement Disorders Society; research grants from the Austrian Science Fund (FWF), The Michael J. Fox Foundation, and International Parkinson and Movement Disorder Society outside of the submitted work. L.J. is an employee of H.Lundbeck A/S and in addition reports research grants from JPND EU Joint Programme–Neurodegeneration Research (67108864), Swedish Research Council for Health, Working Life and Welfare (FORTE), and Sweden's Innovation Agency (VINNOVA). D.O.Å., A.‐K.B., and J.S. are employees of H.Lundbeck A/S. M.F.G. is a full‐time employee of Teva Pharmaceuticals. G.K.W. reports consultancy and lecture fees from AbbVie, AFFiRiS AG, AstraZeneca, Biogen, Biohaven, Inhibicase, Lundbeck, Merz, Ono, Teva, and Theravance and research grants from the Austrian Science Fund (FWF), the Austrian National Bank, the US MSA Coalition, Parkinson Fonds Austria, and International Parkinson and Movement Disorder Society outside of the submitted work. W.P. reports receiving personal fees from AbbVie, AFFiRiS AG, AstraZeneca, BIAL S.A., Boston Scientific, Britannia, Intec, Ipsen, Lundbeck, NeuroDerm, Neurocrine, Denali Pharmaceuticals, Novartis, Orion Pharma, Prexton, Teva, UCB, and Zambon. He receives royalties from Thieme, Wiley Blackwell, Oxford University Press, and Cambridge University Press and grant support from The Michael J. Fox Foundation, European Union FP7 and Horizon 2020 funding programmes.

## Author Roles

(1) Research Project: A. Conception, B. Organization, C. Execution; (2) Statistical Analysis: A. Design, B. Execution, C. Review and Critique; (3) Manuscript: A. Writing of the First Draft, B. Review and Critique.

F.K.: 1A, 1B, 1C, 2A, 2B, 3A, 3B

K.S.: 1A, 1B, 1C, 2A, 2C, 3B

L.J.: 1A, 1B, 1C, 2A, 2C, 3B

D.O.Å.: 1A, 1B, 1C, 2A, 2B, 3A, 3B

A‐K.B.: 1A, 1B, 1C, 2C, 3B

J.S.: 1A, 1B, 1C, 2A, 2B, 3A, 3B

M.F.G.: 1B, 1C, 2C, 3B

G.K.W.: 1A, 1B, 1C, 2A, 2C, 3B

W.P.: 1A, 1B, 1C, 2A, 2C, 3B

## Supporting information


**Table S1** Sensitivity to change in individual Unified Multiple System Atrophy Rating Scale items.

## Data Availability

The data that support the findings of this study are available from Teva Pharmaceutical and the Medical University Innsbruck. Restrictions apply to the availability of these data, which were used under license for this study.
