# Supplementary material for: Sensitivity to Change and Patient‐Centricity of the Unified Multiple System Atrophy Rating Scale Items: A Data‐Driven Analysis
Source: Mov Disord. 2022 Mar 25;37(7):1425–31. doi: 10.1002/mds.28993 (PMC9543676; doi:10.1002/mds.28993)
Supplement: Supplementary file 1 — Table S1 Sensitivity to change in individual Unified Multiple System Atrophy Rating Scale items. [file MDS-37-1425-s001.docx]

# Supplementary Appendix

**EMSA-SG Natural History Study Investigators**

Investigators in this study were: Paolo Barone, Maria Teresa Pellecchia, Niall P. Quinn, Clare J Fowler, Anette Schrag, Nir Giladi, Tanya Gurevich, Karen Ostergaard, Håkan Widner, Wolfgang Oertel, Alberto Albanese, Eduardo Tolosa, Günther Deuschl, Thomas Klockgether, Richard Dodel, Cristina Sampaio, Eldad Melamed, Thomas Gasser, Carlo Colosimo, Olivier Rascol, Wassilios Meissner, François Tison, Felix Geser, Susanne Duerr, Sylvia Boesch, Martin Köllensperger, Vasiliki Koukouni, Christopher J Mathias, Erik Dupont, Christer F Nilsson, Karla Maria Eggert, Francesca del Sorbo, Adriana Cardozo, Helge Hellriegel, Miguel Coelho, Ruth Djaldetti, Christoph Kamm, Giuseppe Meco.

**Rasagiline-for-MSA investigators**

Investigators in this study were: Werner Poewe, Klaus Seppi, Cheryl J Fitzer-Attas, Gregor K Wenning, Sid Gilman, Phillip A Low, Nir Giladi, Paolo Barone, Cristina Sampaio, Eli Eyal, Olivier Rascol, Thomas Chelimsky; Amy Colcher; Praveen Dayalu Neal Hermanowicz; Joseph Jankovic; Grace Liang; Frederick Marshall; Peter Novak; Fernando Pagan; Susan Criswell; Robert Hauser; David Robertson; Stephanie Lessig; Stuart Isaacson; Michel Panisset; Tilak Mendis; Emmanuelle Pourcher; Martin Cloutier; Carlo Colosimo; Alberto Albanese; Giuseppe Orefice; Fabrizio Stocchi; Angelo Antonini; Pietro Cortelli; Eduardo Tolosa; Jaime Kulisevsky; Pablo Mir; Wolfgang Oertel; Albert Ludolph; Günther Deuschl; Daniela Berg; Stefan Lorenzl; Heinz Reichmann; Reinhold Schmidt; Christopher Mathias; David Burn; Huw Morris; Wassilios Meissner; Alain Destee; Miguel Coelho; Ad Hovestadt; Peter Van Domburg; Annamaria Takats; Julia Lajtos; Attila Valikovics; Ruth Djaldetti; Sharon Hassin; Tanya Gurevich; Ilana Shlesinger.

Supplementary Table. Sensitivity to change of individual UMSARS items.

|  | EMSA NHS (Overall) | | | | | EMSA NHS (MSA-P only) | | | | | MSA-Ras | | | | |  |
| --- | --- | --- | --- | --- | --- | --- | --- | --- | --- | --- | --- | --- | --- | --- | --- | --- |
|  |  |  |  | Relative Rank | |  |  |  | Relative Rank | |  |  |  | Relative Rank | |  |
| UMSARS item | Slope | SD | SCS | Slope | Slope (within Subscale) | Slope | SD | SCS | Slope | Slope (within subscale) | Slope | SD | SCS | Slope | Slope (within subscale) | p-Value^1)^ |
| Activities of Daily Living | | | | | | | | | | | | | | | | |
| Item 1: Speech | 0.035 | 0.017 | 2.086 | 14 | 5 | 0.034 | 0.018 | 1.956 | 15 | 5 | 0.063 | 0.047 | 1.328 | 5 | 3 | 0.000 |
| Item 2: Swallowing | 0.045 | 0.013 | 3.368 | 4 | 1 | 0.044 | 0.011 | 3.891 | 5 | 3 | 0.043 | 0.056 | 0.773 | 13 | 8 | 0.000 |
| Item 3: Handwriting | 0.030 | 0.012 | 2.554 | 18 | 8 | 0.028 | 0.010 | 2.713 | 20 | 8 | 0.064 | 0.066 | 0.958 | 4 | 2 | 0.000 |
| Item 4: Cutting Food | 0.038 | 0.022 | 1.687 | 11 | 4 | 0.040 | 0.018 | 2.292 | 9 | 4 | 0.056 | 0.030 | 1.903 | 6 | 4 | 0.000 |
| Item 5: Dressing | 0.045 | 0.025 | 1.775 | 6 | 3 | 0.046 | 0.027 | 1.692 | 4 | 2 | 0.048 | 0.048 | 0.998 | 8 | 6 | 0.573 |
| Item 6: Hygiene | 0.045 | 0.032 | 1.414 | 5 | 2 | 0.048 | 0.028 | 1.728 | 2 | 1 | 0.052 | 0.060 | 0.872 | 7 | 5 | 0.752 |
| Item 7: Walking | 0.031 | 0.018 | 1.731 | 16 | 6 | 0.032 | 0.016 | 2.044 | 17 | 6 | 0.072 | 0.060 | 1.194 | 2 | 1 | 0.000 |
| Item 8: Falling | 0.021 | 0.024 | 0.868 | 23 | 11 | 0.025 | 0.019 | 1.309 | 21 | 9 | 0.009 | 0.034 | 0.274 | 24 | 12 | 0.001 |
| Item 9: Orthostatic Symptoms | 0.015 | 0.010 | 1.531 | 25 | 12 | 0.024 | 0.017 | 1.384 | 23 | 11 | 0.025 | 0.075 | 0.338 | 17 | 10 | 0.508 |
| Item 10: Urinary Function | 0.030 | 0.025 | 1.209 | 17 | 7 | 0.025 | 0.025 | 0.969 | 22 | 10 | 0.044 | 0.057 | 0.774 | 12 | 7 | 0.016 |
| Item 11: Sexual Function | 0.029 | 0.041 | 0.711 | 21 | 9 | 0.031 | 0.045 | 0.681 | 19 | 7 | 0.024 | 0.071 | 0.333 | 19 | 11 | 0.836 |
| Item 12: Bowel Function | 0.022 | 0.020 | 1.132 | 22 | 10 | 0.023 | 0.021 | 1.080 | 24 | 12 | 0.030 | 0.067 | 0.452 | 16 | 9 | 0.419 |
| Motor Examination | | | | | | | | | | | | | | | | |
| Item 1: Facial Expression | 0.046 | 0.011 | 4.057 | 3 | 3 | 0.043 | 0.010 | 4.318 | 7 | 4 | 0.041 | 0.057 | 0.710 | 14 | 6 | 0.003 |
| Item 2: Speech | 0.030 | 0.014 | 2.213 | 19 | 11 | 0.033 | 0.014 | 2.389 | 16 | 11 | 0.047 | 0.054 | 0.873 | 9 | 3 | 0.015 |
| Item 3: Oculomotor Dysfunction | 0.037 | 0.018 | 2.048 | 13 | 9 | 0.040 | 0.007 | 5.578 | 10 | 6 | 0.015 | 0.061 | 0.245 | 22 | 11 | 0.000 |
| Item 4: Tremor at Rest | 0.005 | 0.005 | 1.055 | 26 | 14 | 0.008 | 0.000 | 1703.860 | 26 | 14 | -0.005 | 0.037 | -0.141 | 26 | 14 | 0.000 |
| Item 5: Action Tremor | 0.020 | 0.009 | 2.357 | 24 | 13 | 0.017 | 0.006 | 2.649 | 25 | 13 | 0.006 | 0.029 | 0.200 | 25 | 13 | 0.000 |
| Item 6: Increased Tone | 0.042 | 0.013 | 3.302 | 8 | 5 | 0.043 | 0.009 | 5.071 | 6 | 3 | 0.021 | 0.044 | 0.470 | 20 | 9 | 0.000 |
| Item 7: Rapid Alternating Movement of Hands | 0.037 | 0.012 | 3.086 | 12 | 8 | 0.035 | 0.020 | 1.792 | 13 | 9 | 0.013 | 0.041 | 0.325 | 23 | 12 | 0.000 |
| Item 8: Finger Taps | 0.039 | 0.014 | 2.773 | 10 | 7 | 0.034 | 0.018 | 1.931 | 14 | 10 | 0.025 | 0.042 | 0.594 | 18 | 8 | 0.000 |
| Item 9: Leg Agility | 0.043 | 0.014 | 2.970 | 7 | 4 | 0.042 | 0.015 | 2.857 | 8 | 5 | 0.046 | 0.041 | 1.116 | 10 | 4 | 0.632 |
| Item 10:Heel-Shin Test | 0.052 | 0.020 | 2.549 | 2 | 2 | 0.060 | 0.026 | 2.334 | 1 | 1 | 0.015 | 0.053 | 0.287 | 21 | 10 | 0.000 |
| Item 11: Arising From Chair | 0.054 | 0.027 | 2.010 | 1 | 1 | 0.047 | 0.016 | 2.960 | 3 | 2 | 0.089 | 0.079 | 1.133 | 1 | 1 | 0.000 |
| Item 12: Posture | 0.040 | 0.015 | 2.761 | 9 | 6 | 0.037 | 0.015 | 2.528 | 12 | 8 | 0.033 | 0.026 | 1.264 | 15 | 7 | 0.000 |
| Item 13: Body Sway | 0.035 | 0.020 | 1.721 | 15 | 10 | 0.038 | 0.009 | 4.454 | 11 | 7 | 0.045 | 0.073 | 0.608 | 11 | 5 | 0.869 |
| Item 14: Gait | 0.030 | 0.011 | 2.751 | 20 | 12 | 0.031 | 0.009 | 3.566 | 18 | 12 | 0.068 | 0.035 | 1.940 | 3 | 2 | 0.000 |

^1^p values were estimated using a Mann-Whitney U Test patient’s individual slope assigning patients to either the EMSA NHS (overall) or the MSA-Ras cohort.

Abbreviations: UMSARS, Unified MSA Rating Scale; SD, standard deviation; SCS, sensitivity to change score calculated as slope divided by the SD of the slope.
